# β2-adrenergic receptor agonist counteracts skeletal muscle atrophy and oxidative stress in uremic mice

**DOI:** 10.1038/s41598-021-88438-7

**Published:** 2021-04-28

**Authors:** Takaaki Higashihara, Hiroshi Nishi, Koji Takemura, Hiroshi Watanabe, Toru Maruyama, Reiko Inagi, Tetsuhiro Tanaka, Masaomi Nangaku

**Affiliations:** 1 Division of Nephrology and Endocrinology, The University of Tokyo Graduate School of Medicine, Tokyo, Japan; 2 Department of Biopharmaceutics, Graduate School of Pharmaceutical Sciences, Kumamoto University, Kumamoto, Japan; 3 Division of Chronic Kidney Disease Pathophysiology, The University of Tokyo Graduate School of Medicine, Tokyo, Japan

**Keywords:** Nephrology, Kidney, Kidney diseases

## Abstract

In patients with chronic kidney disease, skeletal muscle dysfunction is associated with mortality. Uremic sarcopenia is caused by ageing, malnutrition, and chronic inflammation, but the molecular mechanism and potential therapeutics have not been fully elucidated yet. We hypothesize that accumulated uremic toxins might exert a direct deteriorative effect on skeletal muscle and explore the pharmacological treatment in experimental animal and culture cell models. The mice intraperitoneally injected with indoxyl sulfate (IS) after unilateral nephrectomy displayed an elevation of IS concentration in skeletal muscle and a reduction of instantaneous muscle strength, along with the predominant loss of fast-twitch myofibers and intramuscular reactive oxygen species (ROS) generation. The addition of IS in the culture media decreased the size of fully differentiated mouse C2C12 myotubes as well. ROS accumulation and mitochondrial dysfunction were also noted. Next, the effect of the β2-adrenergic receptor (β2-AR) agonist, clenbuterol, was evaluated as a potential treatment for uremic sarcopenia. In mice injected with IS, clenbuterol treatment increased the muscle mass and restored the tissue ROS level but failed to improve muscle weakness. In C2C12 myotubes stimulated with IS, although β2-AR activation also attenuated myotube size reduction and ROS accumulation as did other anti-oxidant reagents, it failed to augment the mitochondrial membrane potential. In conclusion, IS provokes muscular strength loss (uremic dynapenia), ROS generation, and mitochondrial impairment. Although the β2-AR agonist can increase the muscular mass with ROS reduction, development of therapeutic interventions for restoring skeletal muscle function is still awaited.

## Introduction

Sarcopenia is classically defined as a decline in the skeletal muscle mass, strength, and endurance. Nowadays, particularly low muscle strength is underscored as a critical characteristic and dysfunction to be treated^1^. Although it has been well studied in the context of ageing, a growing amount of epidemiological evidence has revealed a high incidence of sarcopenia in elderly patients with chronic kidney disease (CKD) as kidney function deteriorates^2–7^. The importance of uremic sarcopenia lies in its impact on mortality and morbidity, including fractures that affect quality of life for CKD patients^8^, cardiovascular events^9^, and overall survival^10^.

In addition to multiple contributors pinpointed by previous studies^11–13^, inappropriate accumulation of uremic toxin may yield uremic sarcopenia. Uremic toxins are usually filtered and excreted by healthy kidneys but are accumulated in the body when kidney function has deteriorated. Among the uremic toxins, the protein-bound uremic toxins, such as indoxyl sulfate (IS) and p-cresyl sulfate, are hardly ever removed by hemodialysis because of their high affinity to serum albumin^14,15^ and are considered directly harmful to visceral systems such as the kidney^16–18^, cardiovascular system^19,20^, and immune system^21^. IS as a representative uremic toxin is generated in the liver via the metabolism of indole absorbed in the gut^22^. Accumulation of IS also exerts negative effects on myoblast proliferation and myotube size in vitro and skeletal muscle mass in vivo^23–27^. In addition, IS induced metabolic alterations resulting in mitochondrial dysfunction and ATP shortage in muscle cells^28^. However, skeletal muscle functioning after direct administration of IS has not been well documented in mice. Also, previous reports demonstrated that secondary sarcopenia due to debilitating diseases showed a decrease, predominantly in fast-twitch glycolytic or oxidative-glycolytic fibers^29–36^. Yet, it is not documented which type of myofiber suffers the most damage in uremic sarcopenia.

Although exercise and dietary modification may benefit patients with uremic sarcopenia^11,13,37^, pharmacological interventions have not been optimized. β2-adrenergic receptor (β2-AR) agonist may be promising as it can trigger skeletal muscle hypertrophy via promotion of protein synthesis^38^. Among them, clenbuterol is one of those that have been studied as a potential remedy for muscle wasting and has been reported to reduce rodent muscle atrophy due to neuromuscular damages such as denervation^39,40^ or hindlimb suspension^41^. A small clinical trial with patients who had brachial plexus injuries support its benefits for denervation-induced muscle mass reduction^42^. However, it has not been well exploited in the context of metabolic and malnutritional diseases such as CKD, so we hypothesize that β2-AR signaling in skeletal muscle counteracts uremic sarcopenia.

Our study aims to elucidate whether the uremic toxin, IS, directly exerts a negative effect on not only the size but also the functioning of skeletal muscle, to determine which type of myofiber is vulnerable, and to explore the mechanism and potential treatment for IS-induced muscle atrophy and dysfunction.

## Results

### IS injection in mice decreases skeletal muscle strength

**Figure 1:**
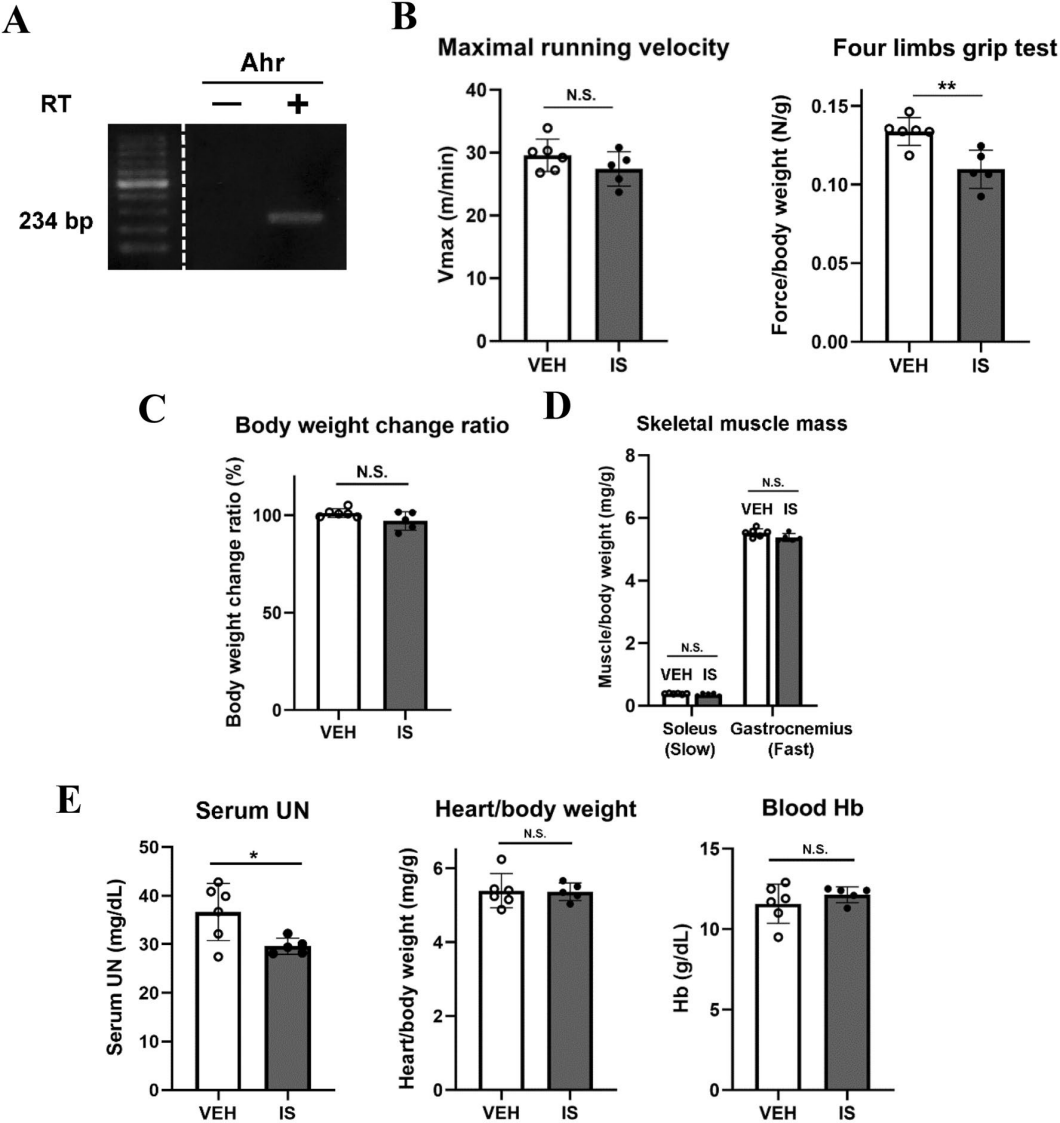
IS administration caused skeletal muscle weakness and a tendency toward muscle atrophy in mice after unilateral-nephrectomy. (**A**) Expression of the endogenous receptor of indoxyl sulfate (IS) and the aryl hydrocarbon receptor (Ahr) was confirmed with electropheresis for PCR products of mouse gastrocnemius (GC) muscle cDNA. PCR products without reverse transcriptase (RT) were loaded as negative controls. The figure was cropped to show regions of interest. Full-length of the gel is shown in the supplementary Fig. 1. (**B**) Maximal running velocities (Vmax) measured in a treadmill running exhaustion test (left) and grip strengths from the four limbs grip test (right) are shown. Mice underwent unilateal nephrectomies one week prior to treatment with vehicle (VEH) or IS for 7 days. (**C**) Body weight change ratios before and after 7 days of treatment, and (**D**) skeletal muscle wet weight/body weight of soleus and GC muscles after one week of treatment are shown. (**E**) Serum urea nitrogen (UN, left), heart/body weight (middle), and blood hemoglobin (Hb, right) after one week of treatment are shown. N = 5–6 mice per group. Error bars denote SEM. * p < 0.05, ** p < 0.01, N.S.; no significance, bp; base pairs.

To evaluate the toxic effect of IS on the skeletal muscle, we injected the mice intraperitoneally with IS. Since normal kidneys excrete IS nearly completely, the mice underwent unilateral nephrectomy 7 days before intraperitoneal IS injection. After IS injection every 24 h for a week, high-performance liquid chromatography (HPLC) analysis revealed IS accumulation in the gastrocnemius (GC) muscle of 2,697 ± 1,153 pmol/mg protein in mice injected with IS versus 10.9 ± 2.2 pmol/mg protein in mice injected with vehicle control (n = 3 for each). We also confirmed expression of the aryl hydrocarbon receptor (AhR), an endogenous receptor bound by IS^43^, in the skeletal muscle tissue (Fig. 1A). Then, we evaluated whether mouse skeletal muscle function was influenced by IS treatment. A four limbs grip strength test indicated that the instantaneous strength was impaired in the mice injected with IS, while treadmill endurance and capacity was not affected (Fig. 1B). In those mice, the body weight (Fig. 1C) and the weight of the GC and the soleus muscle (Fig. 1D) showed no significant reduction. In this condition, the other organ dysfunctions which could possibly have conferred poor exercise performance, such as kidney failure^16,17^, anemia^18^ and cardiac hypertrophy^19^, were undetectable (Fig. 1E).

### IS exerts an injurious effect primarily on fast-twitch myofibers

**Figure 2:**
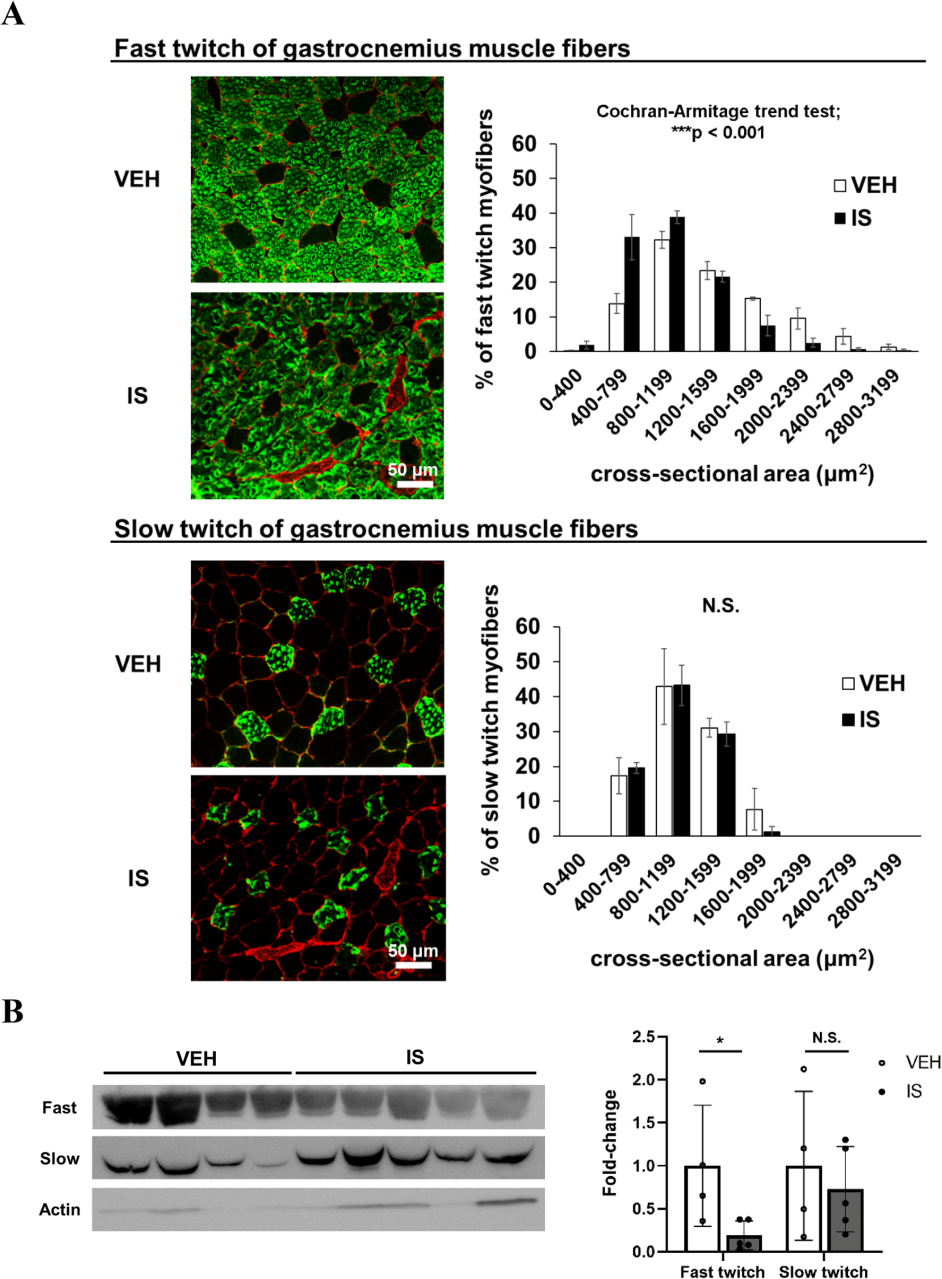
IS administration caused a decrease in predominantly fast-twitch muscle fiber. (**A**, **B**) Immunohistochemical analysis of (**A**) fast (top, *green*) and slow (bottom, *green*) myosin heavy chain (MHC) isoforms counterstained with laminin (*red*) was performed for mouse gastrocnemius (GC) muscle cryosections obtained from mice with a unilateral nephrectomy after one-week treatment of vehicle (VEH) or indoxyl sulfate (IS) (left). The scale bar is 50 μm. The cross-sectional area and size distribution of fast and slow myofibers was quantitated (right). As a statistical analysis, a Cochran-Armitage trend test for fiber size distribution was performed. N = 4 mice per group. Error bars denote SEM. (**B**) Western blotting analysis of the fast and slow MHC isoforms was performed in GC muscle (left). Actin was utilized as a loading control. Quantitative densitometric data from western blots are shown (right). N = 4–5 mice per group. A full-length view of the membrane is shown in the supplementary Fig. 2. Error bars denote SEM. * p < 0.05, N.S.; no significance.

To explore the mechanism of IS-induced muscle weakness, we evaluated the changes in fast-twitch myofibers playing a pivotal role in instantaneous muscle strength. Although composed predominantly of fast-twitch myofibers, mouse GC muscle is a muscle with mixed fiber types. However, immunofluorescent staining of mouse GC muscle with fast- (type 2) and slow-twitch (type 1) myosin heavy chain antibodies revealed that IS induced a decrease predominantly in cross-sectional areas of fast-twitch muscle fiber (Fig. 2A). We further observed a predominant decrease in protein expression of fast-twitch myosin heavy chains (MHC) by western blotting (Fig. 2B). These results indicate that the injurious effect of IS is noted mainly in fast-twitch myofibers.

### Protein synthesis is suppressed in atrophic skeletal muscles of mice injected with IS

**Figure 3:**
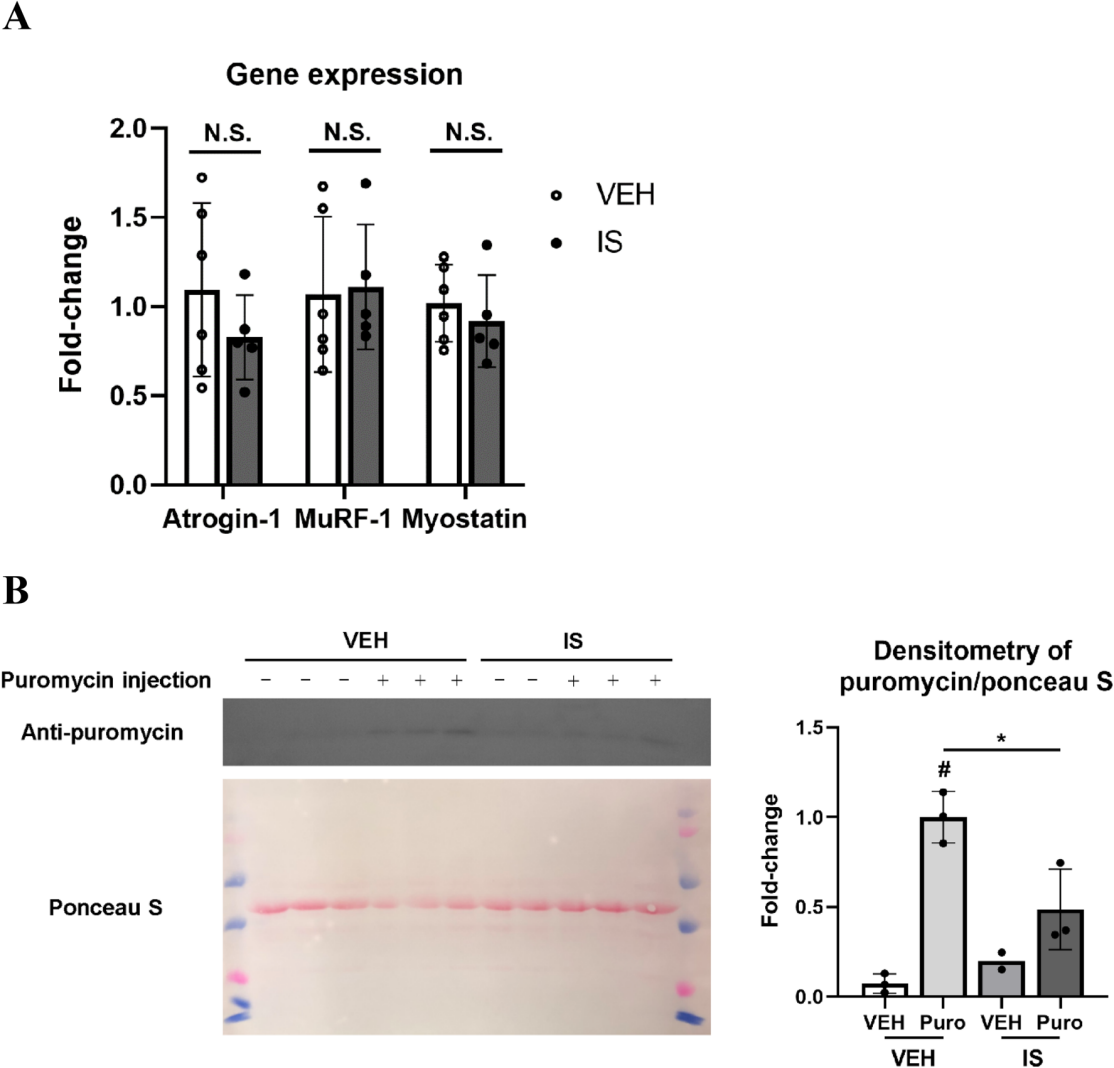
IS administration did not change the gene expression of proteolysis-related genes but suppressed protein synthesis (puromycin-labeled peptides). (**A**) qPCR analysis for mRNA expression of atrogin-1, MuRF-1, and myostatin in gastrocnemius (GC) muscle of unilaterally nephrectomized mice treated with vehicle (VEH) or indoxyl sulfate (IS). N = 5–6 mice per group. Error bars denote SEM. N.S.; no significance. (**B**) Western blotting using an anti-puromycin antibody was performed for analysis of GC muscle protein synthesis (puromycin-labeled peptides). The ponceau S staining was examined as a loading control. Quantitative densitometric data from western blots are shown (right). N = 2–3 mice per group. A full-length view of the membrane is shown in the supplementary Fig. 3. Error bars denote SEM. * p < 0.05, Puro; puromycin. #; p < 0.001 compared to VEH without IS treatment.

Muscle quantity is determined by the homeostatic balance of protein synthesis and proteolysis^44^. To elucidate the mechanism of muscle atrophy in mice injected with IS, we first evaluated atrophy related gene expression associated with classic ubiquitin–proteasome pathways, such as myostatin and atrogenes (atrogin-1, and MuRF-1), which are involved in the pathomechanism of skeletal muscle atrophy in sarcopenia^45^. In IS treated mouse GC muscle, these atrophic gene expressions were not increased (Fig. 3A). Therefore, we conducted in vivo experiments using puromycin assays for analyzing protein synthesis, the surface sensing of translation technique^46,47^. Western blotting analysis revealed that the expression of peptides labeled with puromycin was noted only in mice injected with puromycin, as anticipated, and was decreased in mice treated with IS (Fig. 3B), indicating suppression of muscle protein synthesis in these mice. Therefore, as a mechanism for reduction of skeletal muscle mass in the current model, IS treatment may restrict protein synthesis rather than promote proteolysis via atrogene-related ubiquitination.

### Clenbuterol increases mouse muscle mass but does not improve weakness induced by IS

**Figure 4:**
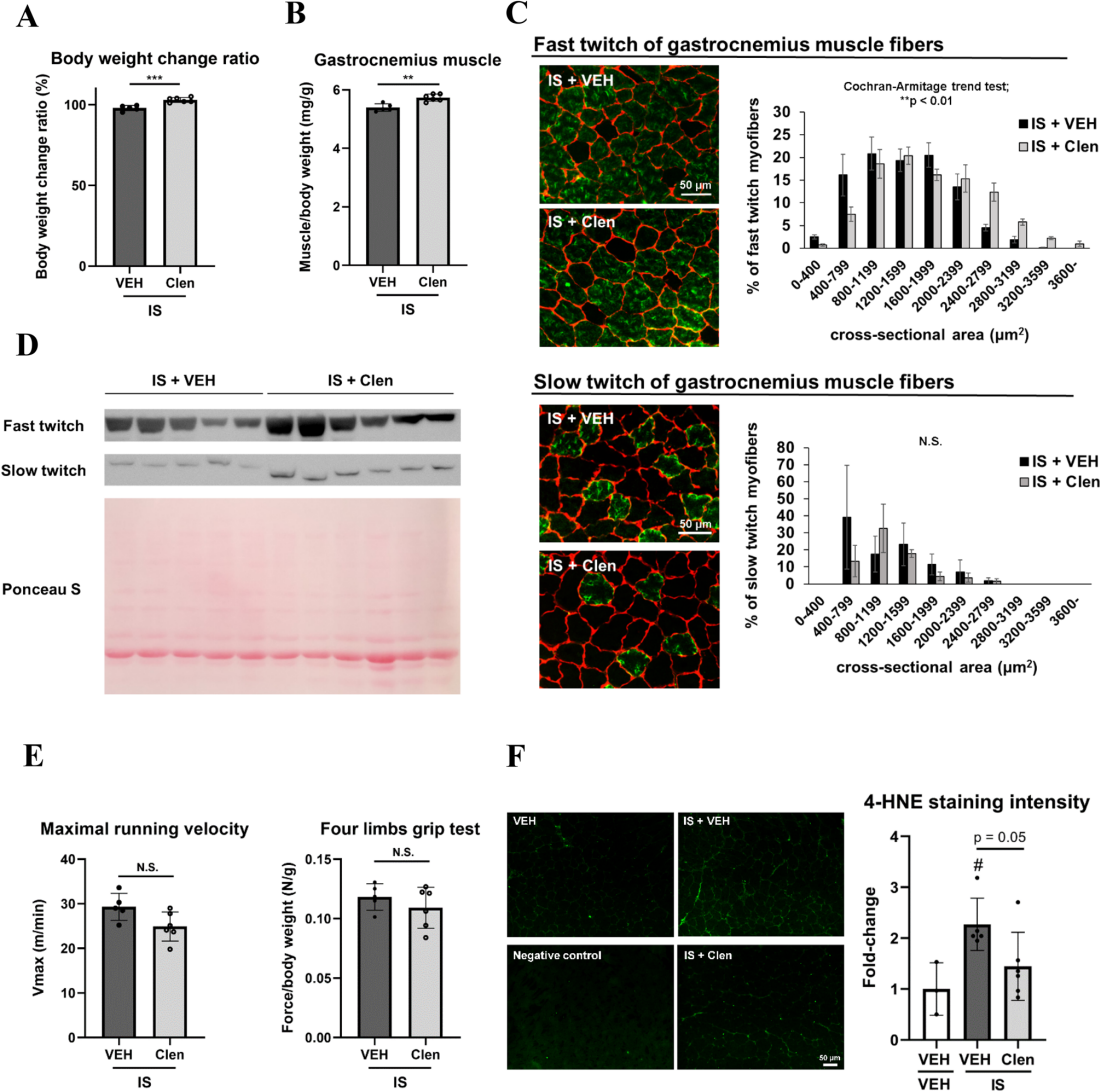
β2-AR agonist, clenbuterol treatment increased GC muscle mass and protein expression of MHC even with IS treatment, but could not reverse muscle weakness. (**A**) The ratio of mouse body weight change before and after one-week treatment of indoxyl sulfate (IS), and IS plus clenbuterol is shown. (**B**) The ratio of gastrocnemius (GC) muscle wet weight over the total body weight of mice after one-week treatment is shown. (**C**) Immunohistochemical analysis of fast (top, green) and slow (bottom, green) myosin heavy chain (MHC) isoforms counterstained with laminin (red) was performed for mouse GC muscle cryosections obtained from those 2 groups. The scale bar is 50 μm. The cross-sectional area and size distribution of fast and slow myofibers was quantitated (right). As a statistical analysis, a Cochran-Armitage trend test for fiber size distribution was performed. N = 5–6 (fast twitch) and 3 (slow twitch) mice per group. Error bars denote SEM. (**D**) Western blotting analysis of the fast and slow MHC isoforms in GC muscle obtained from those 2 groups was performed (top). Ponceau S staining was examined as a loading control (bottom). A full-length view of the membrane is shown in the supplementary Fig. 4. (**E**) Maximal running velocity (Vmax) measured in treadmill running exhaustion tests (left) and grip strength measured in four limbs grip tests (right) are shown. (**F**) 4-HNE staining on mouse GC muscle cryosections after one week of treatment with vehicle or clenbuterol and IS (left). The same cryosections without adding the first anti-4-HNE antibody were also examined as a negative control. The scale bar is 50 μm. Signal intensity was quantitated (right). N = 3–6 mice per group. Error bars indicate SEM. ** p < 0.01, *** p < 0.001. #; p < 0.05 compared to vehicle (VEH) without IS treatment. Clen; clenbuterol. N.S.; no significance.

Next, to explore potential treatments for uremic sarcopenia, we focused on the ergogenic β2-AR agonists allegedly used for livestock in several countries^48,49^. Moreover, since β2-AR agonists induce slow-to-fast MHC isoform transition^50^, a β2-AR agonist could theoretically be a promising way to ameliorate skeletal muscle mass reduction and dysfunction in our model where predominantly fast-twitch myofibers have deteriorated. Indeed, clenbuterol treatment increased not only body weight (Fig. 4A) but GC muscle mass (Fig. 4B) in mice treated with IS. Furthermore, clenbuterol treatment led to an increase in cross-sectional areas (Fig. 4C) and MHC protein expression (Fig. 4D) of fast-twitch muscle fiber predominantly. However, intriguingly, clenbuterol treatment could not restore IS-induced muscle weakness (Fig. 4E).

IS, as a representative uremic toxin, induces reactive oxygen species (ROS) accumulation in tissues and organs^51–53^. Previous reports illustrated that ROS plays a pivotal role in skeletal muscle atrophy associated with ageing or cardiovascular diseases^54–56^. Therefore, we postulated that ROS is accumulated in mouse GC muscle treated with IS which might be attenuated by clenbuterol treatment. Immunofluorescent staining with 4-hydroxynonenal (4-HNE) revealed that lipid peroxidation was enhanced in mice treated with IS that was blunted by clenbuterol treatment (Fig. 4F).

### Clenbuterol does not restore mitochondrial function in cultured myotubes stimulated with IS

**Figure 5:**
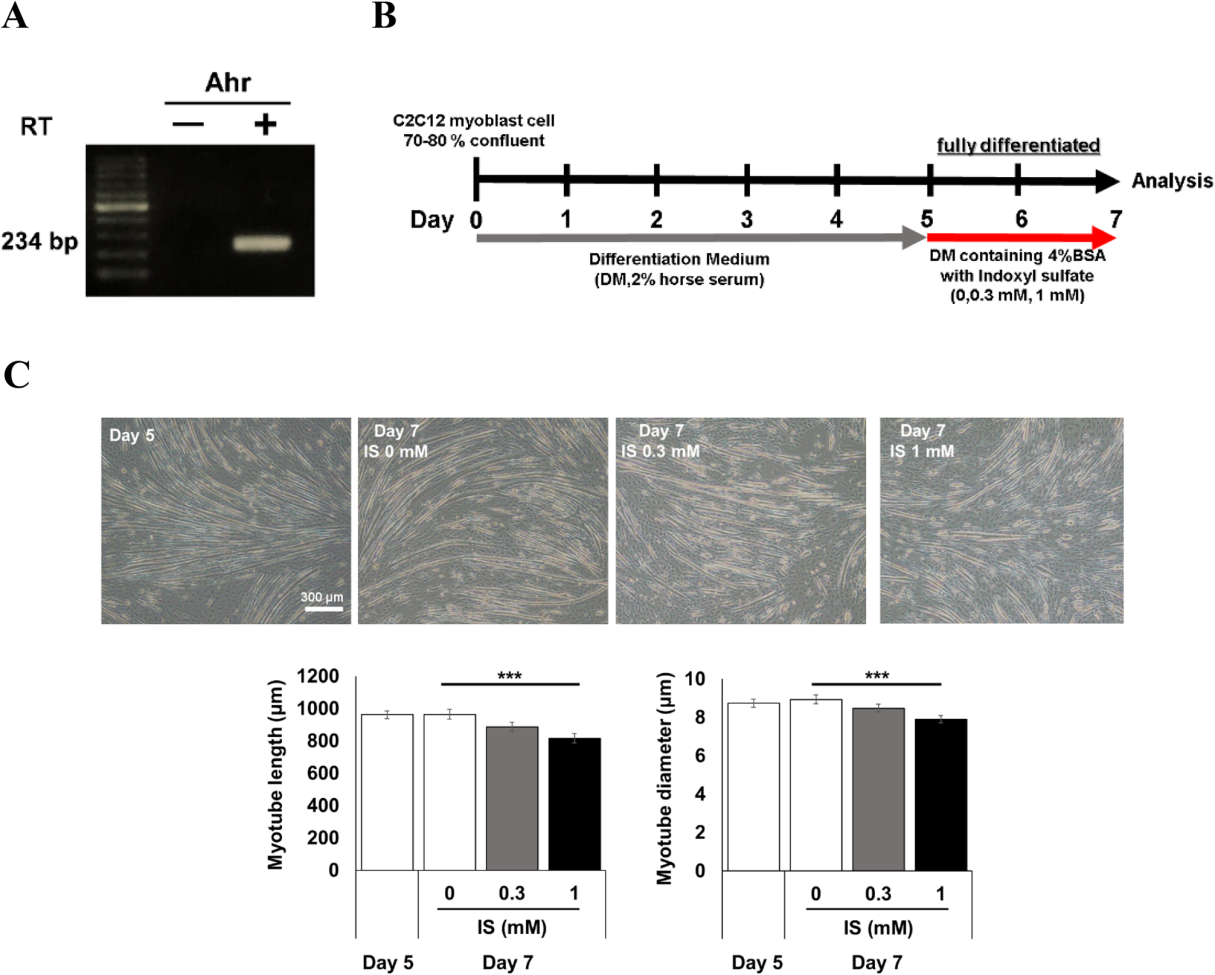
Ahr receptor was detected in C2C12 myotubes and IS administration caused cell size reduction in fully differentiated C2C12 myotubes. (**A**) Expression of Ahr was confirmed with electropheresis for PCR products of mouse C2C12 myotube cDNA. PCR products without reverse transcriptase (RT) was loaded as a negative control. (**B**) Differentiated C2C12 myotubes were stimulated with indoxyl sulfate (IS) together with BSA as IS binds to albumin with a high affinity in vivo. (**C**) Representative micrographs of C2C12 myotubes stimulated with IS at 0 mM, 0.3 mM and 1 mM for 2 days were shown (top). The scale bar is 300 μm. Myotube morphology was quantitatively evaluated in terms of the length (lower left) and the diameter (lower right). N = 100 and 200 myotubes per group, respectively. Error bars indicate SEM. *** p < 0.001.

After confirming that C2C12 myotubes express AhR, an endogenous IS receptor (Fig. 5A), Since IS in the bloodstream forms a complex that binds to albumin, the absence of proper albumin quantities could result in a high concentration of free IS and induce exaggerated biologic responses with an overestimated toxic effect, as detailed elsewhere^15^. Therefore, we co-administered IS and albumin to the culture media (Fig. 5B). This treatment decreased the length and diameter of fully differentiated myotubes (Fig. 5C).

**Figure 6:**
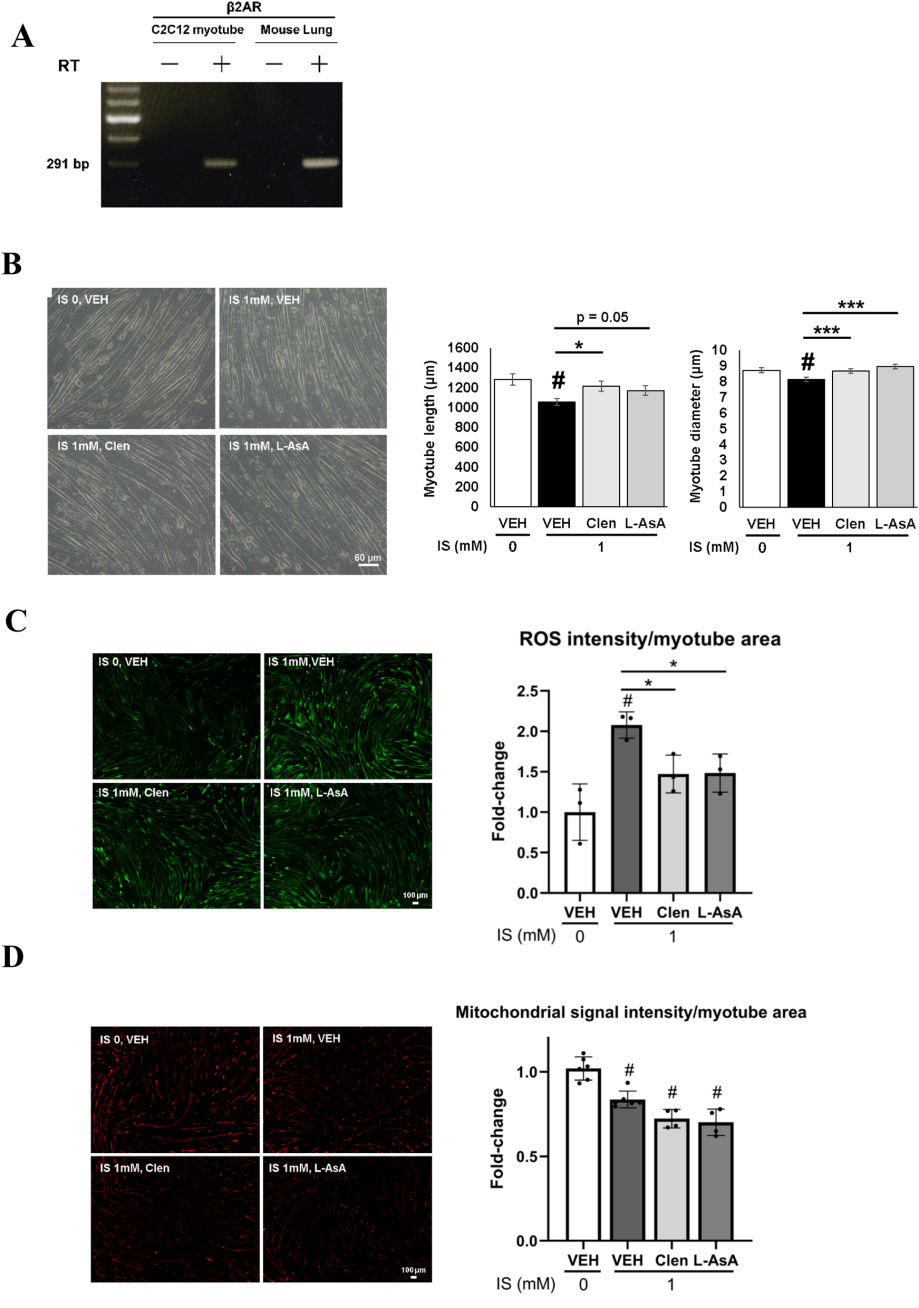
IS induced cell size reduction and increased ROS accumulation that were suppressed by treatment with L-ascorbic acid and clenbuterol. (**A**) Expression of β2-AR was confirmed with electropheresis for PCR products of mouse C2C12 myotube cDNA. PCR products without reverse transcriptase (RT) was loaded as a negative control. (**B**) Representative micrographs of C2C12 myotubes stimulated with vehicle (VEH), IS, IS plus clenbuterol (Clen), and IS plus L-ascorbic acid (L-AsA) for 24 h were shown (left). The scale bar is 60 μm. Myotube morphology was quantitatively evaluated in terms of the length and the diameter (right). N = 100 and 200 myotubes per group, respectively. (**C**) Cellular ROS accumulation was evaluated with CM-H2DCFDA staining intensity in C2C12 myotubes treated with vehicle (VEH), IS, IS plus Clen, and IS plus L-AsA for 24 h (left). The scale bar is 100 μm. Signal intensity was quantitated (right). N = 3 per group. (**D**) Mitochonrial membrane potential was evaluated with MitoTracker Red (left). Staining intensity in C2C12 myotubes treated with VEH, Clen, and L-ascorbic acid (L-AsA) for 1 h. followed by incubation with or without IS for another 1 h was quantitated (right). The scale bar is 100 μm. N = 4–6 per group. Error bars indicate SEM. * p < 0.05, *** p < 0.001, #; p < 0.05 compared to VEH without IS treatment.

Next, after confirmation of β2-AR expression in C2C12 myotube (Fig. 6A), we evaluated whether clenbuterol treatment may modulate the effect of IS on myotubes. Thus, clenbuterol and anti-oxidant L-ascorbic acid ameliorated IS-induced myotube size reduction (Fig. 6B). Stimulation with IS also induced cellular ROS accumulation in myotubes, which was confirmed by its partial cancellation after L-ascorbic acid treatment (Fig. 6C). Another anti-oxidant, N-Acetyl-L-cysteine, also attenuated the myotube size reduction as well as ROS accumulation that are both induced by IS (Supplementary Fig. 5), suggesting that ROS scavenging contributes to the increase in cell size. Intriguingly, treatment of C2C12 myotubes with clenbuterol partially eliminated the intracellular ROS accumulation that had been induced by IS stimulation (Fig. 6C).

Then, we focused on mitochondrial dysfunction which reportedly centers IS toxicity in the skeletal muscle and causes uremic sarcopenia^7,23,28^. Thus, MitoTracker Red staining of the C2C12 myotube reveled that IS treatment decreased the mitochondrial membrane potential that is critical for the energy production needed for muscle contraction (Fig. 6D). Then, clenbuterol treatment failed to restore the reduction of mitochondrial membrane potential in myotubes (Fig. 6D).

## Discussion

Our data demonstrates that IS administration to mice following unilateral nephrectomy is sufficient to deteriorate four limbs grip strength while exercise endurance and capacity were spared. We also found that skeletal muscle size reduction mainly occurs in fast-twitch myofibers that play an important role in instantaneous muscle strength and its quantity is associated with the risk of falls and hip fractures resulting in long hospitalizations and increased mortality^57–60^. In ageing sarcopenia, physiological and morphological changes in skeletal muscle are characterized by an overall decline in the size and number of skeletal muscle fibers, mainly the fast-twitch muscle fibers^59^. That applies also to secondary sarcopenia due to malnutrition and debilitating diseases such as cancer, diabetes, sepsis, and infection^29–34,36^. Therefore, preventing fast-twitch muscle fiber atrophy may be one of the treatment targets for uremic sarcopenia.

In the current study, we injected mice with IS for seven days after unilateral nephrectomy. Enoki et al. also clarified skeletal muscle atrophy with elevated atrogene expression in unilaterally nephrectomized mice injected with lower doses of IS for a longer period (100 mg/kg body weight for 12-weeks)^23^. Moreover, the muscle wet weight decreased in both GC muscle and soleus muscle rich in slow-twitch muscle fibers. Although our animal model failed to show the same results regarding GC muscle, the fast-twitch MHC protein expression and instantaneous muscle strength were significantly decreased. These changes may represent the characteristics of “dynapenia,” which was proposed in 2008 to define the age-related loss of muscle strength rather than muscle mass reduction^61^. Dynapenia is typical for age-related muscle weakness characterized by reduction in fast-twitch (type 2) muscle fibers and increased intramuscular adipose tissues^62,63^. Furthermore, a past study showed that dynapenia is more prevalent than sarcopenia in pre-dialysis CKD patients, and 50% of the dynapenic patients were in early stage of CKD^64^. Therefore, dynapenic changes with the decrease in predominantly fast-twitch muscle via impaired protein synthesis seen in our protocol might be the very early changes of IS-induced sarcopenia, followed by the whole fiber type reduction via proteolysis in the chronic phase.

The gene expressions of myostatin and atrogenes such as atrogin-1 and MuRF-1 did not change in the current model although these genes were originally found to be up-regulated by fasting and inactivity in early phases of muscle wasting^65,66^. Increased expression of these genes was also reported after subtotal nephrectomy or long-term treatment of uremic toxin in experimental animals, accompanied by significant body weight loss^23,24,67^. However, the current animal model with IS injection did not develop significant loss of body weight and food-intake, thus explaining the discrepancy. Also, to be noted, it remains controversial whether atrogin-1 and MuRF-1 signaling is operative in muscle wasting during ageing. Atrogin-1 and/or MuRF1 mRNA levels are reportedly unchanged in aged muscle of human^68^ or even decreased in that of rats^69,70^. Instead, our in vivo experiments show that IS suppressed skeletal muscle protein synthesis, rather than proteolysis, due to atrogene-related ubiquitination. Past reports demonstrated that C2C12 myotubes treated with IS show decreased insulin-stimulated protein expression of phospho-S6K1^28^. Also, in the mouse CKD model with subtotal nephrectomy, a deficiency in the nucleolar protein 66, selectively, in the skeletal muscle counteracts degradation of protein synthesis independently of myostatin signaling^71^. These findings are in line with our current concept that impairment of skeletal muscle protein synthesis contributes to uremic sarcopenia.

To explore therapeutic intervention for the IS toxic effects described above, a β2-AR agonist might have been suitable because it reinforces mainly fast MHC isoforms and up-regulates the Akt-mTOR protein synthesis pathway^50^. In our study, the β2-AR agonist increased the mouse GC muscle mass rich in fast-twitch myofibers and protein expression of MHC. Surprisingly, clenbuterol treatment also attenuated the ROS accumulation caused by IS just as anti-oxidant L-ascorbic acid treatment did, which has not been reported before. However, expression of anti-oxidant enzymes such as catalase and glutathione peroxidase 1 was not increased by clenbuterol treatment (data not shown). Therefore, the mechanism for clenbuterol role in diminishing IS-induced ROS accumulation still needs to be elucidated.

In a recent clinical guideline, low muscle strength was highlighted as a key characteristic of sarcopenia and a restoration of skeletal muscle function is recognized as one of the therapeutic goals^1^. In this regard, clenbuterol treatment alone is still suboptimal as we could not find significant improvement of skeletal muscle strength in uremic mice, which has also been reported elsewhere in humans^72,73^, possibly for the following reasons. First, as shown in our C2C12 experiments, clenbuterol was not sufficient for potentiation of the mitochondrial membrane potential that was impaired by IS. The result is in line with the current concept that mitochondrial dysfunction plays a pivotal role in uremic sarcopenia^74^. Second, IS treatment may directly affect muscle contraction independently of muscle mass. Skeletal muscle contraction requires calcium-dependent cellular responses, and the ER has a pivotal role in maintaining calcium concentration^75^. Moreover, IS induces ER stress on human proximal tubular cells and mouse C2C12 myocytes^17,76^. Therefore, IS-induced mitochondrial dysfunction and ER stress may disturb energy metabolism and calcium-dependent responses on myotube cells, respectively, and result in skeletal muscle weakness. Third, we observed the capacity of clenbuterol to cause amplification of fast-twitch fibers, which is consistent with previous reports^77,78^. This uneven alteration of fiber type may be disadvantageous for skeletal muscle performance. Interestingly, when clenbuterol administration is accompanied by low endurance training^79^ or electrical stimulation^72^, the slow- to fast-twitch fiber transformations are suppressed and then muscle performance are potentiated.

From a clinical viewpoint, since it is a decongestant and bronchodilator and is approved for treatment of bronchial asthma in several countries, drug repurposing for uremic sarcopenia is deemed not unrealistic. However, clenbuterol might promote arrhythmia and cardiomegaly particularly after long-term administration^73^, so, optimally, may not be used for patients with CKD who have a high risk of cardiovascular diseases^80^.

In conclusion, a representative albumin-bound form of uremic toxin, IS, directly impairs skeletal muscle strength and leads to dynapenic phenotype in mice. Clenbuterol treatment may be effective in increasing skeletal muscle mass via reduction of ROS accumulation. For uremic myopathy, work toward development of a new drug to intervene against impaired muscle strength needs to be continued.

## Materials and methods

### Reagents

Clenbuterol (C5423), L-ascorbic acid (A92902), N-Acetyl-L-cysteine (A7250), anti-actin (A2066), anti-skeletal slow myosin (M8421), anti-skeletal fast myosin (M4276), anti-puromycin (clone 12D10, MABE343, Millipore), and anti-laminin polyclonal antibody (AB19012, Millipore) were purchased from Sigma-Aldrich (St Louis, MO, USA). IS was purchased from Nacalai Tesque (#19,208–04, Kyoto, Japan). The other reagents were described in each experiment.

### HPLC analysis

IS levels in the plasma and gastrocnemius were measured by an HPLC method as described previously^24^. In brief, plasma or a gastrocnemius homogenate (homogenized in distilled water) was mixed with acetonitrile (1:9, v/v for the sample) and centrifuged at 12,000 g for 10 min. The supernatant was collected and was loaded to HPLC with 10 μL for the plasma sample or the tissue homogenate. The HPLC system consisted of Jasco PU-4180 pump and Jasco FP-4020 fluorescence spectrophotometer (Tokyo, Japan). A CAPCELL PAK C18 MG II column (SHISEIDO, Tokyo, Japan) was used for the stationary phase. The mobile phase consisted of 0.2 M acetate buffer (pH4.5)–acetonitrile (3:1, v/v) for IS. The flow rate was 1.0 mL/min. IS was detected by means of a fluorescence monitor with excitation/emission wavelengths set to 280 and 375 nm, respectively.

### Animals

C57BL/6J female mice (7–8 weeks of age, 19.36 ± 0.15 g body weight) were purchased from CLEA Japan (Tokyo, Japan), and housed on a 12 h day/night cycle. Mice were anesthetized using an isoflurane-oxygen mixture (3% induction and 1.5–2.5% maintenance) and the left kidney was removed. At 1 week after surgery, mice were administrated with IS (600 mg/kg body weight, ip; intraperitoneal injection, everyday) for 7 days. IS was dissolved in phosphate-buffered saline (PBS). The control mice were administrated with PBS for 7 days at the same volume. In another protocol, clenbuterol (2 mg/kg body weight) was also intraperitoneally injected 30 min before the IS injection. The mouse body weight was measured every day. Treadmill exhaustion tests and four limbs grip strength tests were performed 12 h before sacrifice. At the end of the study, mice were anesthetized with the isoflurane-oxygen mixture (5% induction), and then mouse body weight was measured, and blood, kidney, heart, and skeletal muscles (soleus and gastrocnemius) were harvested. The concentrations of blood urea nitrogen (BUN) in mice blood serum were measured with commercial test kits (Wako Pure Chemical Industries, Ltd., Japan). Hemoglobin level was measured with an automatic blood cell counter. The animal experiments were carried out in accordance with the ARRIVE guidelines.

### Treadmill exhaustion tests

Treadmill exhaustion tests were executed utilizing TMS-8B (Melquest Ltd, Japan). Mice were kept running individually on separated lanes with a shock grid (stimulatory shock of 0.3 mA) at the rear end. Before the test, mice were acclimated to treadmill running with the speed increased gradually 1 m/min to a final speed of 10 m/min for 10 min. After acclimation, treadmill speed was gradually increased by 3 m/min every 2 min. When mice remained in the shock-grid area for more than 10 s, the mice were regarded as exhausted and promptly removed from lanes. The result was recorded as maximal velocity at the withdrawal time. In this paper, the result was represented as maximal velocity (Vmax).

### Four limbs grip strength test

Peak force of the four limbs grip was measured using a grid attached to an isometric force transducer (IMADA, Toyohashi, Japan). Mice were allowed to grasp the metal grid with all four limbs. The maximum force exerted was recorded. This test was repeated 5 times and an average score was calculated. To achieve validation, all grip strengths (N, Newtons) were normalized to body weight (g, gram).

### Muscle fiber-type analysis

GC muscles were harvested from C57BL/6J mice, embedded in the Tissue-Teck O.C.T compound (#4583, Sakura), and rapidly frozen in isopentane fully cooled by liquid nitrogen. GC muscle cryosections (5 μm-thick slices) were blocked in PBS containing 0.3% Triton X-100 and M.O.M blocking reagent (Vector Laboratories, USA) for 1 h at room temperature. Slices were then incubated overnight at 4 °C with primary antibodies, such as the anti-laminin antibody (1:500), and anti-skeletal fast (1:500) or slow myosin (1:1,000) antibodies in the blocking solution with M.O.M. protein concentrate (Vector Laboratories) as previously described^81^. Section slices were incubated with secondary antibodies, such as Alexa Fluor 488 anti-mouse IgG antibody (1:2,000) and TRITC-conjugated anti-rabbit IgG antibody (1:40) in PBS containing 0.3% Triton-X-100 for 1 h at room temperature. Image acquisition was performed with an inverted fluorescence microscope, BZ-X710 (Keyence, USA). The fiber cross-sectional area was measured for approximately 200 adjacent muscle fibers in each section for each mouse using ImageJ software (NIH, Bethesda, USA) as described elsewhere^82^.

### Analysis for skeletal muscle protein synthesis

Skeletal muscle protein synthesis was evaluated in vivo by the surface sensing of translation technique as previously described^46,47^. Under anesthesia, 0.04 μmol puromycin/g body weight (Sigma-Aldrich, St Louis, MO) diluted in PBS was intraperitoneally injected. Then, the GC muscle was removed 15 min. after puromycin administration. After homogenization, the supernatant was collected and used for western blotting.

### Quantitative reverse-transcriptase PCR analysis

Total RNA was extracted using RNAiso Plus (Takara Bio Inc., Shiga, Japan) according to the manufacture’s protocol. The cDNA was synthesized using Prime Script RT master mix (TaKaRa Bio Inc.). To confirm expression of AhR and β2-AR, the primers shown in Table 1 were used. Quantitative real-time RT-PCR analysis of myostatin, atrogin-1, MuRF-1, and GAPDH was performed in a CFX96 System (Bio-Rad Laboratories. Inc., USA) with THUNDERBIRD SYBR QPS-201 (Toyobo Co. Ltd., Japan). The primers used are shown in Table 2. The threshold cycle (Ct) values for each gene amplification were normalized by subtracting the Ct value calculated for GAPDH.

**Table 1:** Primers in PCR.

Target gene	Forward	Reverse
Ahr	5′-CACAGAGTTAGACCGCCTGG-3’	5′- TTCAGCGCCTGTAACAAGAAC-3’
β2-AR	5′-CTGGTTGGGCTACGTCAACT-3’	5′- CTTCCTTGGGAGTCAACGCT-3’

**Table 2:** Primers in real time RT-PCR.

Target gene	Forward	Reverse
MuRF-1	5′-GACTCCTGCAGAGTGACCAAG-3’	5′-CTTCTACAATGCTCTTGATGAGC-3’
Atrogin-1	5′-CAGAGAGGCAGATTCGCAAG-3’	5′-GGTGACCCCATACTGCTCTC-3’
Myostatin	5′-CCAGGCACTGGTATTTGGCA-3’	5′-AAGGGATTCAGCCCATCTTCTC-3’
GAPDH	5′-CATGGCCTTCCGTGTTCCTA-3’	5′-CCTGCTTCACCACCTTCTTGAT-3’

### Western blotting analysis

The whole-cell lysate was prepared by using RIPA buffer (50 mM Tris–HCL (pH 8.0), 150 mM sodium chloride, 0.5% sodium deoxycholate, 0.1% sodium dodecyl sulfate (SDS), 1.0% TritonX-100) containing a protease inhibitor cocktail (cOmplete Mini, Roche, Basel, Switzerland) or Halt protease and phosphatase inhibitor cocktails (Thermo Fisher Scientific). SDS sample buffer containing dithiothreitol (DTT, final concentration 2.5 mM) was added to 15–30 μg of total protein per lane and was separated with 8% or 10% SDS–polyacrylamide gel electrophoresis. After electrophoresis, the proteins were transferred onto polyvinylidene difluoride membrane (GE Healthcare, UK), and then we assessed gel loading either by Ponceau S staining (Sigma-Aldrich) or by actin-antibody. The membrane was blocked with 5% skim milk for 30 min at room temperature followed by incubating with the first antibody overnight at 4 ℃ and then with the secondary antibodies such as horseradish peroxidase-conjugated goat anti-rabbit antibody (1:10,000, Bio-Rad) or anti-mouse antibody (1:10,000, Bio-Rad) for 45 min at room temperature. ECL Plus Western blot substrate (Thermo Fisher Scientific) was used for detecting the signals. The intensity of bands was quantitated by ImageJ software (NIH, Bethesda, USA).

### Cell culture

Murine C2C12 myoblast cells obtained from American Type Culture Collection (ATCC, Manassas, VA, USA) were cultured in low-glucose Dulbecco’s Modified Eagle’s Medium (DMEM; D6046, Sigma-Aldrich) containing 10% fetal bovine serum (FBS; Sigma-Aldrich) and 100 unit/ml penicillin and 100 μg/ml streptomycin. After reaching semi-confluence (70–80%) the culture medium was changed to a differentiation medium, DMEM containing 2% heat-inactivated horse serum (Sigma-Aldrich) to induce differentiation to myotubes. IS was dissolved in serum-free DMEM, and cells were stimulated with the 4% albumin containing medium with IS.

### Myotube length and diameter measurement

C2C12 myoblast cells were seeded in a 6-well or 12-well plate and cultured in a differentiation medium as described above. When cells reached full differentiation after 5 days, the cultured medium was replaced with DMEM containing 4% bovine serum albumin (either with or without IS, clenbuterol (1 μM), L-ascorbic acid (100 μM) and N-Acetyl-L-cysteine (1 mM)) and cultured for 1 or 2 more days. The medium with IS and reagents were changed every day. Cell morphology was observed with microscopy (Olympus, Inc.). Myotube length and diameters were measured in at least 100 to 200 myotubes using ImageJ software (National Institute of Health (NIH), Bethesda, MD, USA).

### ROS accumulation assay

For cultured cells, CM-H2DCFDA (C6827, Thermo Fisher Scientific) fluorescent dye was utilized, mainly to detect hydrogen peroxide. C2C12 myoblast cells were seeded in a 6-well or 12-well plate and cultured as described above. After becoming fully differentiated, myotubes were co-incubated with 4% albumin and IS (1 mM) in the presence or absence of clenbuterol (1 μM), L-ascorbic acid (100 μM) and N-Acetyl-L-cysteine (1 mM) for 24 h. After treatment, myotubes were washed with serum-free DMEM and incubated with CM-H2DCFDA (1 μM) conjugated serum-free DMEM for 30 min at 37 ℃ under 5% CO2. After removing the medium, myotubes were washed with PBS twice. Then, image acquisition was performed with an inverted fluorescence microscope, BZ-X710 (Keyence, USA). The intensity of CM-H2DCFDA staining was quantitated by ImageJ software (NIH, Bethesda, USA).

For animal tissue specimens, immunofluorescent staining with anti-4-HNE antibody (20 μg/mL; JaICA, Shizuoka, Japan) was utilized to detect lipid peroxidation derived from ROS generation. The overall methods are the same as in the muscle fiber-type analysis described above, but we changed the thickness of the GC muscle cryosections (10 μm-thick slices) and used Alexa Fluor 488 anti-mouse IgG antibody (1:2,000) as a secondary antibody. Image acquisition was performed with an inverted fluorescence microscope, BZ-X710 (Keyence, USA). The intensity of 4-HNE staining was quantitated by ImageJ software (NIH, Bethesda, USA).

### MitoTracker Red staining

For cultured cells, MitoTracker Red (M7521, Thermo Fisher Scientific) fluorescent dye to detect viable mitochondrial membrane potential was utilized. C2C12 myoblast cells were seeded in a 12-well plate and changed to a differentiation medium as described above. After 5 days, having reached full differentiation, the cultured medium was changed to DMEM containing 4% bovine serum albumin. Differentiated myotubes were incubated with or without clenbuterol and L-ascorbic acid for 1 h, and then incubated with or without IS for another hour. Myotubes were washed with serum-free DMEM and incubated with the MitoTracker Red dye conjugated serum-free DMEM for 30 min at 37 ℃ under 5% CO2. After removing the medium, the myotubes were fixed with 4% paraformaldehyde for 5 min, and then washed with PBS twice. Images were acquired with an inverted fluorescence microscope, BZ-X710 (Keyence, USA) and quantitated by ImageJ software (NIH, Bethesda, USA).

### Statistical analysis

All data were expressed with the means and error bars representing SEM. Statistical analysis was performed using a two-tailed Student t-test. A Cochran-Armitage trend test was performed to examine the change in the frequency distribution of the cross-sectional area of muscle fiber. A p-value < 0.05 was considered statistically significant.

### Ethics statement

All animal experiments were approved by the ethics committee of the University of Tokyo Graduate School of Medicine and performed following the guidelines for the care and use of laboratory animals.

## Supplementary Information

Supplementary Information
