# Supplementary material for: β2-adrenergic receptor agonist counteracts skeletal muscle atrophy and oxidative stress in uremic mice
Source: Sci Rep. 2021 Apr 28;11:9130. doi: 10.1038/s41598-021-88438-7 (PMC8080640; doi:10.1038/s41598-021-88438-7)
Supplement: Supplementary file 1 — Supplementary Information [file 41598_2021_88438_MOESM1_ESM.docx]

**Supplementary Figure 1**

**
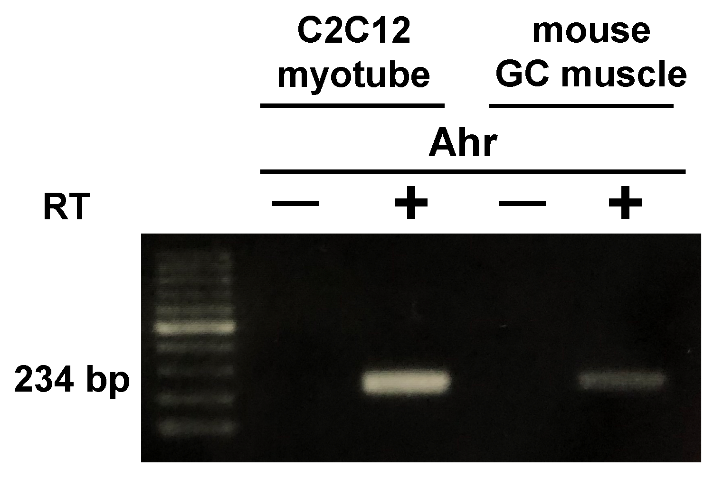
**

**Supplementary Figure 1. The Ahr is expressed in the mouse C2C12 myotube and mouse GC muscle.**

The expression of the endogenous receptor of indoxyl sulfate (IS), the aryl hydrocarbon receptor (Ahr), was confirmed with electropheresis for reverse transcription PCR products of mouse C2C12 myotubes and mouse gastrocnemius (GC) muscle cDNA. PCR products without reverse transcriptase (RT) were loaded as negative controls.

**Supplementary Figure 2**

**
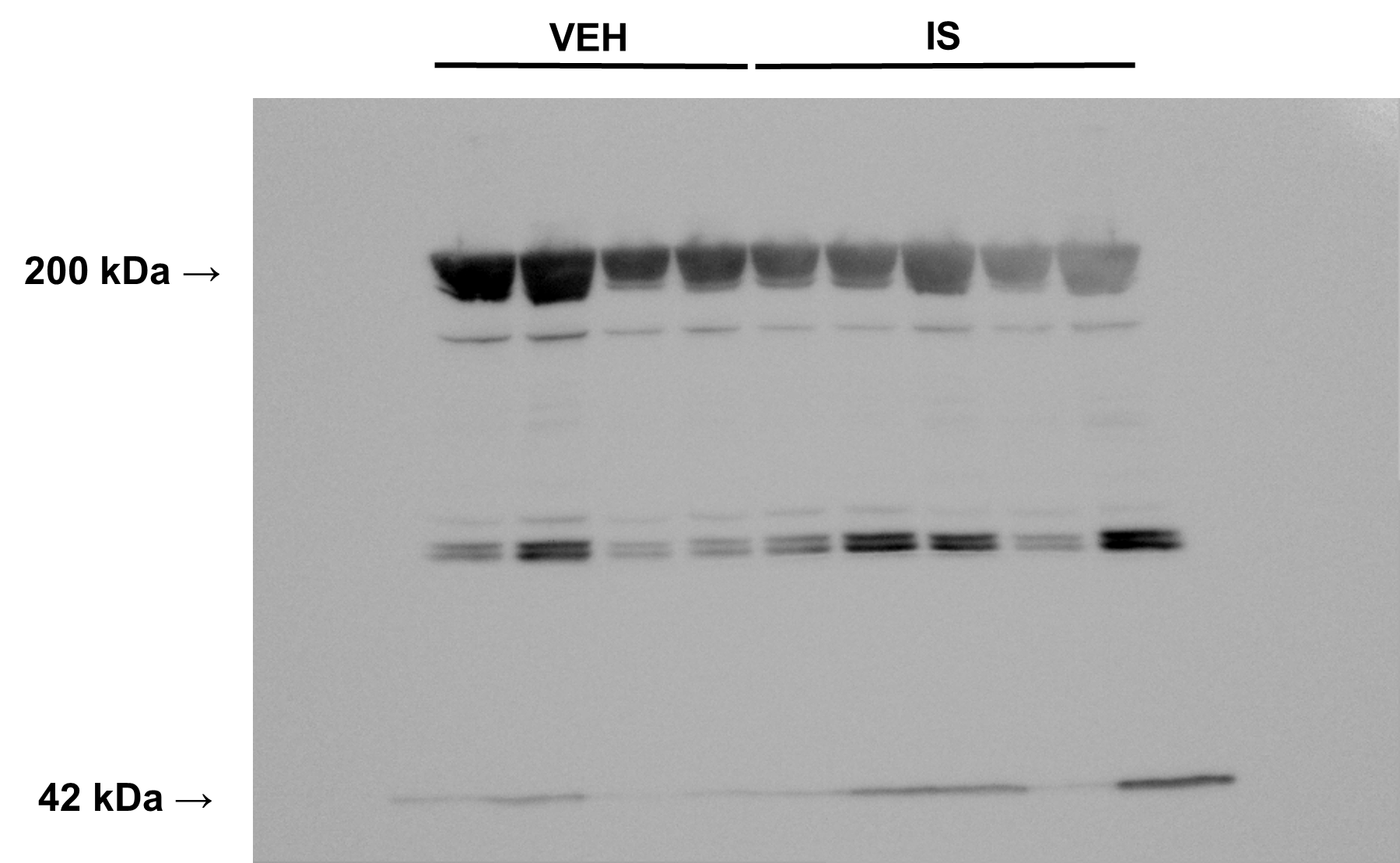
**

**
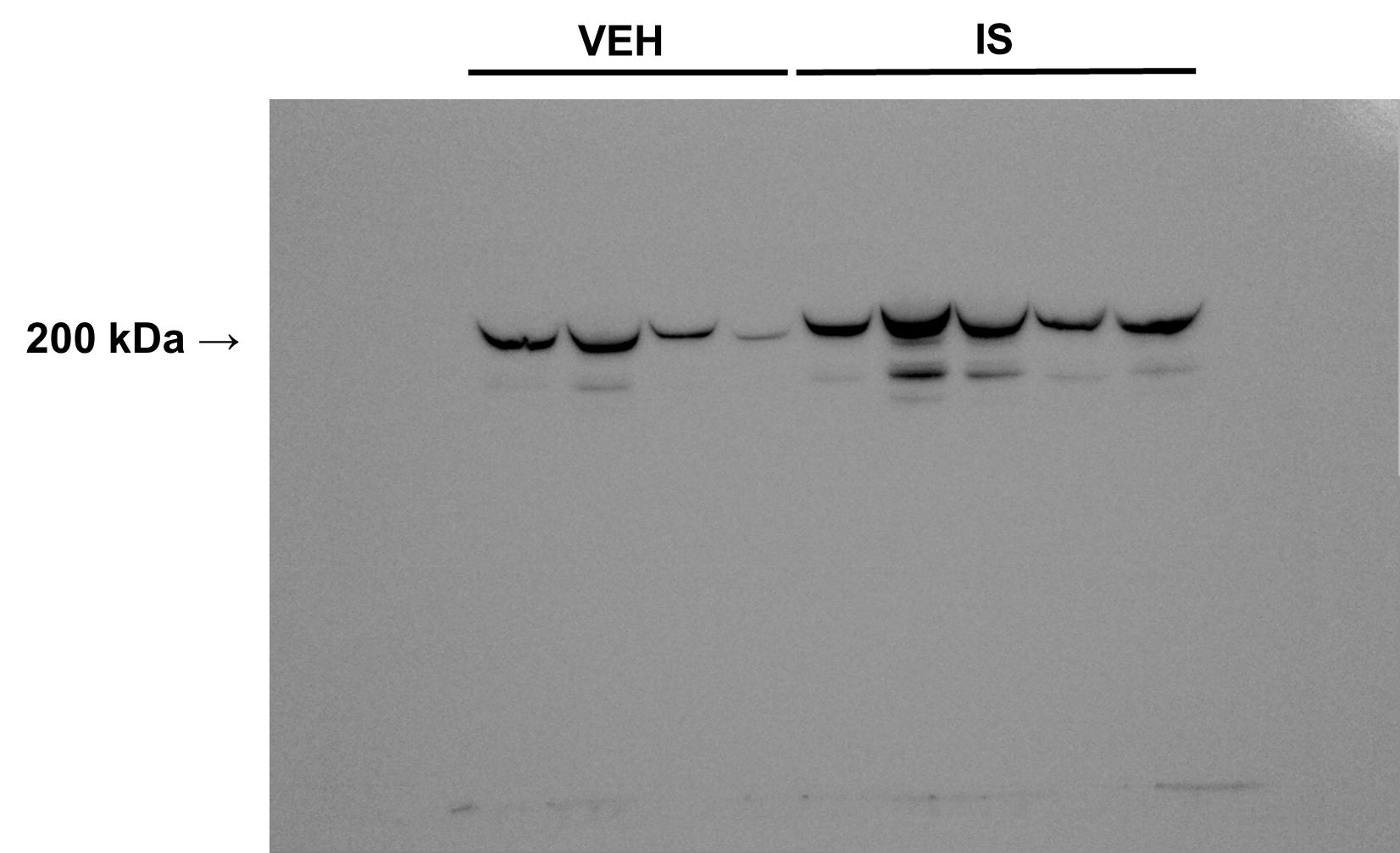
**

**Supplementary Figure 2. IS administration caused a decrease of predominantly fast-twitch muscle fiber.**

Western blotting analysis of the fast (top, 200 kDa) and slow (bottom, 200 kDa) MHC isoforms in GC muscle　 was performed. Actin was utilized as a loading control (top, 42 kDa). In a top panel, the two separated membranes were developed at the same time. N = 4-5 mice per group

**Supplementary Figure 3**

**
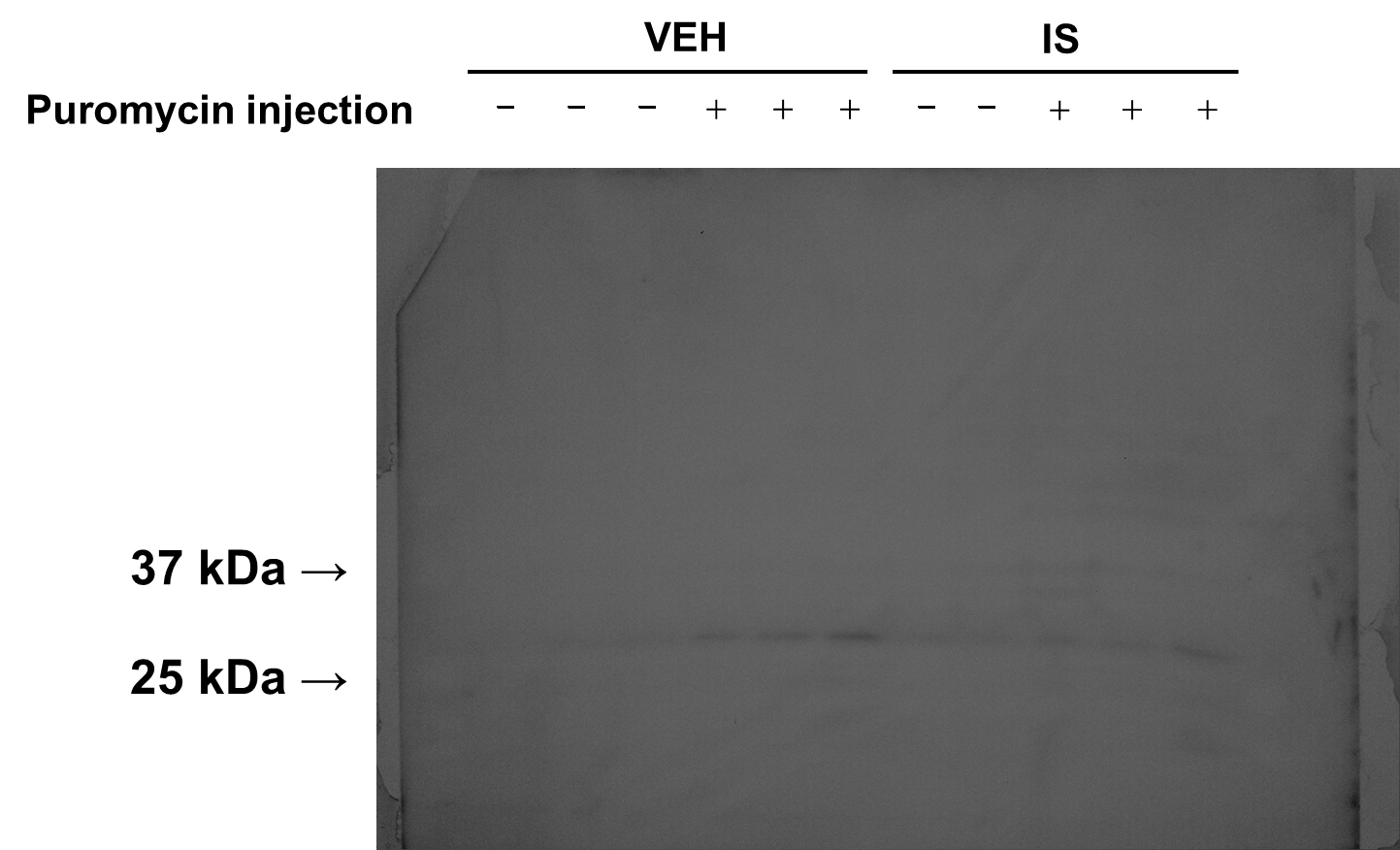
**

**
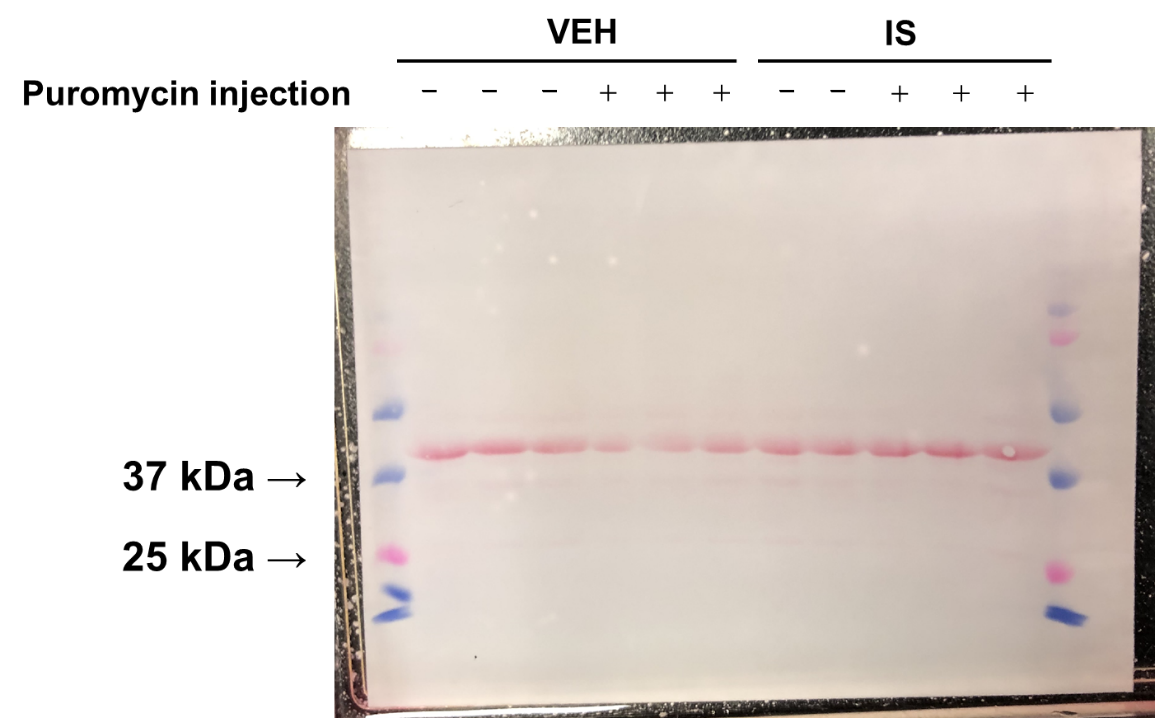
**

**Figure 3. IS administration suppressed protein synthesis (puromycin-labeled peptides).**

Western blotting using an anti-puromycin antibody was performed for analysis of GC muscle protein synthesis (top). The ponceau S staining was examined as a loading control (bottom). N = 2-3 mice per group.

**Supplementary Figure 4**

**A**


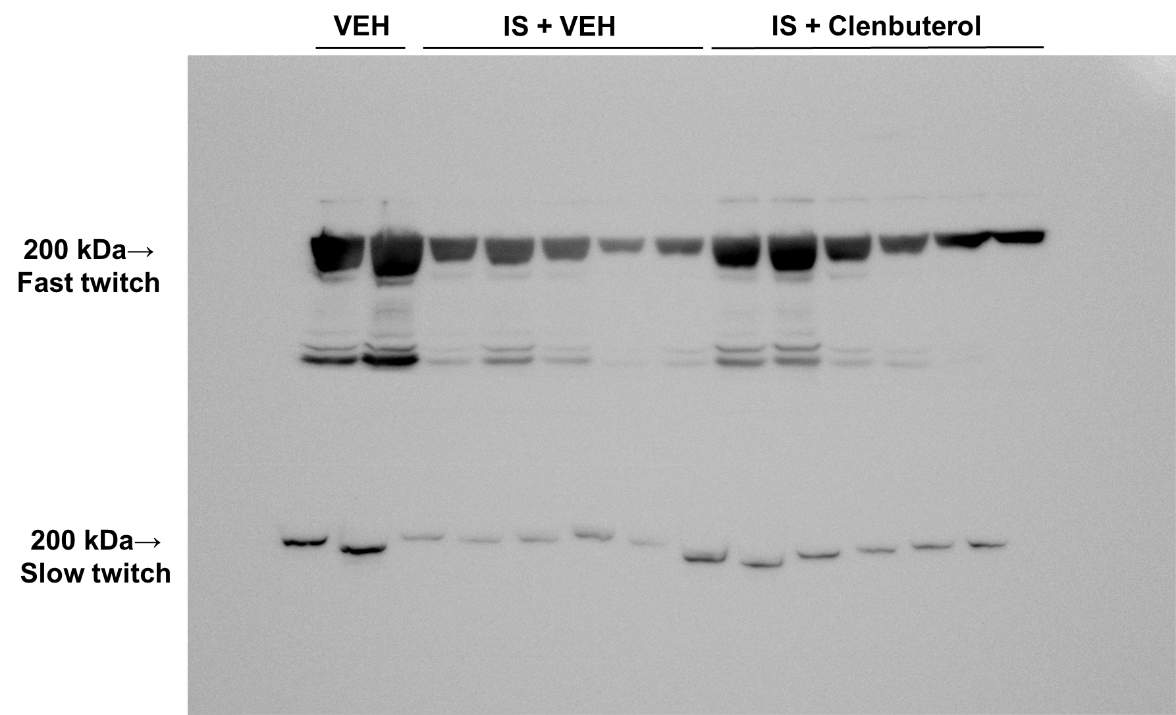


**B**


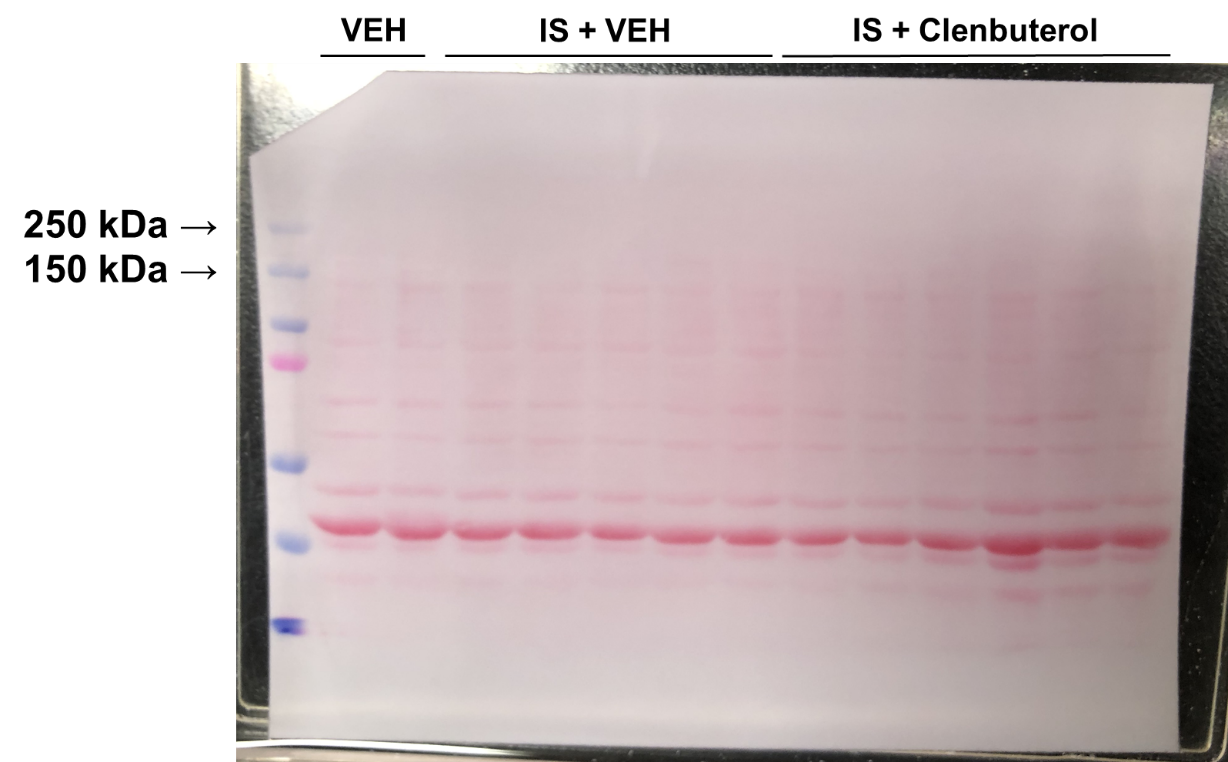


**Supplementary Figure 4. β2-AR agonist, clenbuterol treatment increased GC muscle protein expression of MHC even with IS treatment.**

(A) Western blotting analysis of the fast (top) and slow (bottom) MHC isoforms in GC muscle obtained from 2 groups with small number of VEH group was performed. In a top panel, the two separated membranes were developed at the same time. (B) Ponceau S staining was examined as a loading control (bottom). N = 2-6 mice per group. GC; gastrocnemius, VEH; vehicle, IS; indoxyl sulfate, Clen; clenbuterol.

**Supplementary Figure 5**

**A**


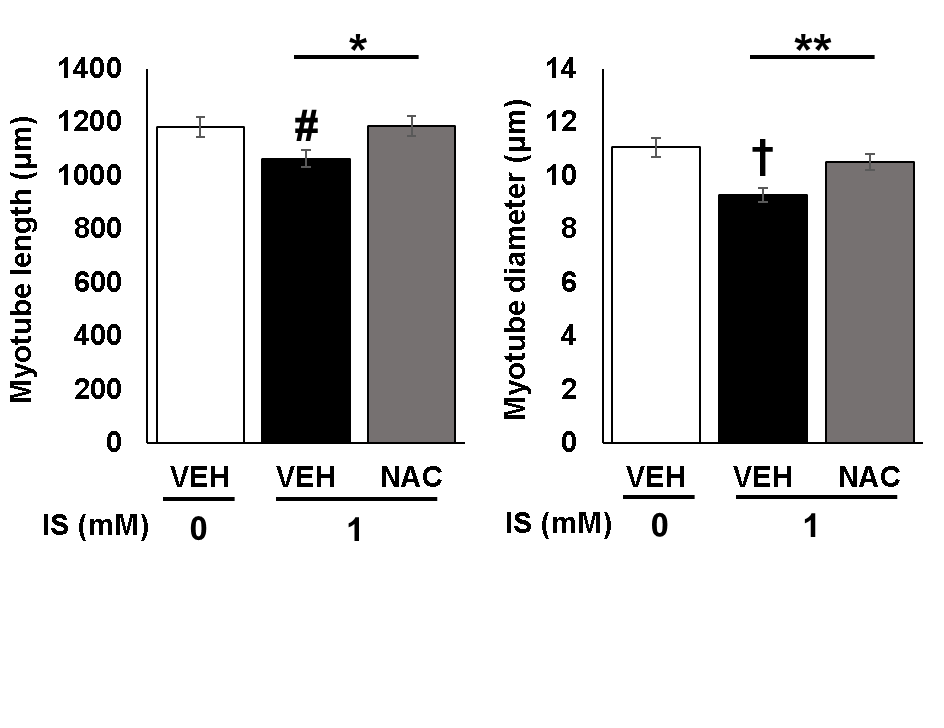

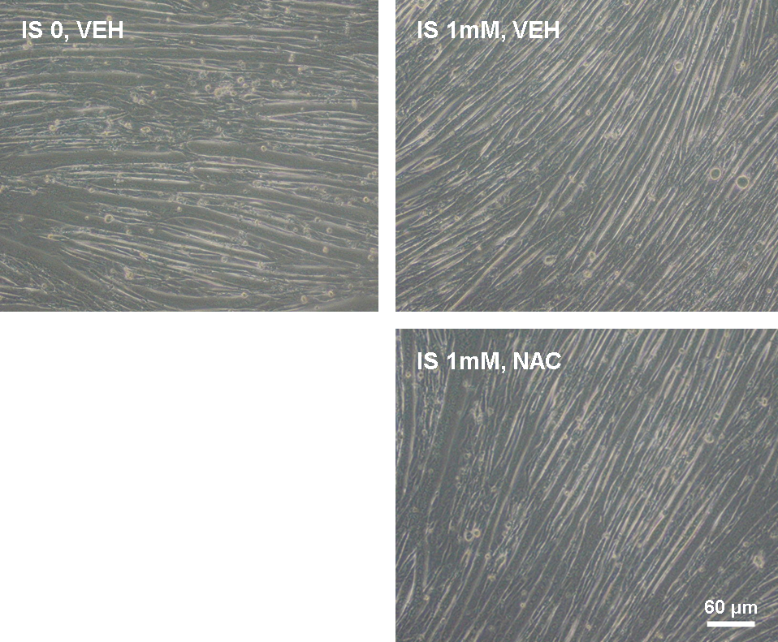


**B**


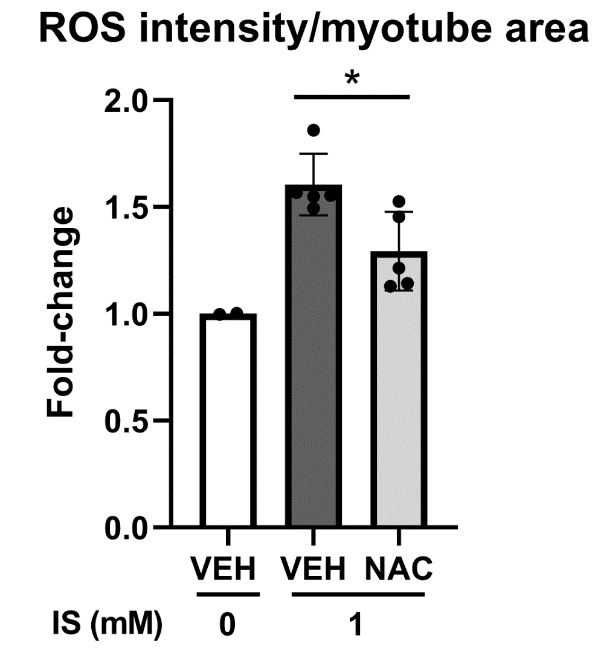


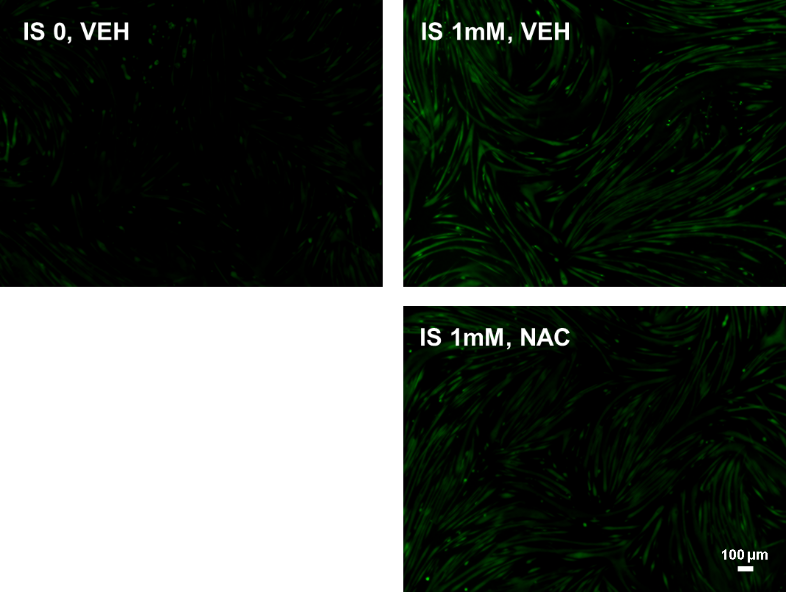


**Supplementary Figure 5. IS induced cell size reduction and increased ROS accumulation that were suppressed by treatment with N-Acetyl-L-cysteine.**

(A) Representative micrographs of C2C12 myotubes stimulated with vehicle (VEH), indoxyl sulfate (IS), IS plus N-Acetyl-L-cysteine (NAC) for 48 hrs were shown (left). Experiments were repeated twice. The scale bar is 60 μm. Myotube morphology was quantitatively evaluated in terms of the length and the diameter (right). N = 100 myotubes per group. (B) Cellular ROS accumulation was evaluated with CM-H2DCFDA staining intensity in C2C12 myotubes treated with VEH, IS, IS plus NAC for 24 hrs. (left). The scale bar is 100 μm. Signal intensity was quantitated (right). N = 2-5 per group. Error bars indicate SEM. * p < 0.05, ** p < 0.01, #; p < 0.05 compared to VEH without IS treatment, †; p < 0.001 compared to VEH without IS treatment.
